# Determinants of prelacteal feeding practices among mothers of children aged less than 24 months in Ile-Ife Southwest Nigeria: a community cross-sectional study

**DOI:** 10.11604/pamj.2019.34.172.17642

**Published:** 2019-12-03

**Authors:** Tolulope Ogundele, Olorunfemi Akinbode Ogundele, Adedokun Isaac Adegoke

**Affiliations:** 1 Department of Paediatrics, University of Medical Sciences, Ondo City, Ondo State, Nigeria; 2 Department of Community Medicine, University of Medical Sciences Ondo City, Ondo State, Nigeria; 3 Department of Obstetrics and Gynaecology, University of Medical Sciences Teaching Hospitals Complex, Ondo City, Ondo State, Nigeria

**Keywords:** Determinants, prelacteal feeding, community, mothers, children

## Abstract

**Introduction:**

Prelacteal feeding remains an obstacle in achieving the best breastfeeding practices in the country. The growing poor breastfeeding practices are made worse by the continued engagement of the communities in prelacteal feeding practices. This study aimed at assessing the determinants of prelacteal feeding among mothers of children aged less than 24 months in Ile-Ife Southwest, Nigeria.

**Methods:**

A community-based cross-sectional study that employed quantitative and qualitative methods. Two hundred and fifty-five (255) mother with children aged 0-23 months were recruited into the study using a multistage sampling technique. SPSS version 20 was used for data analysis Descriptive statistics, bivariate and multivariable logistic regression analysis was done.

**Results:**

In this study, 26.3% of children were given prelacteal feeds. Glucose water (46.3%), sugar water (25.4%) and infant formula (17.9%) were commonly given prelacteal feeds. On multivariate analysis initiating breastfeeding after one hour (Adjusted Odds Ratio (AOR): 2.74, 95% CI 1.43, 5.23), not attending antenatal clinic (AOR = 2.52, 95% CI 1.05, 5.33), delivery via caesarian section 52% (AOR = 1.52, 95 % CI 1.10, 6.34) were associated with increased odds of giving prelacteal feeds. Delivery attended by health professional 25% (AOR = 0.75. 95% CI 0.42, 0.97), highest wealth quintiles 21% (AOR =0.79, 95 % CI 0.51, 0.94) were associated with lowers odds of giving prelacteal feeds.

**Conclusion:**

Prelacteal feeding was prevalent in the study community and associated with community, individual and health service-related factors. Intervention that strengthens individual and community access to appropriate health information and maternal health services is vital in reducing prelacteal feeding practices.

## Introduction

Prelacteal feeding has been linked with poor breastfeeding and health outcomes. It is a documented contributing factor to suboptimal breastfeeding practices. Prelacteal feeding is harmful to the health of newborns because it limits infant's frequency of sucking and exposes them to increased risk of infection [1, 2]. According to a recent study, infants who received prelacteal feeding were more likely to be stunted and wasted [1]. The practice of giving prelacteal feeds has been found to be a key determinant of breastfeeding initiation and early cessation of optimal breastfeeding [3]. Prelacteal feed fills the newborn's stomach quickly and interferes with sucking and makes breastfeeding more difficult to establish. This can, in turn, reduce breast milk production and provide an opportunity for early discontinuation of exclusive breastfeeding, and in effect encourage the practice of prelacteal feeding [4]. Prelacteal feeding, therefore, remains a major challenge to optimal breastfeeding and adequate infant nutrition [5]. To achieve child health-related sustainable development goals (SDGs) in the country and also retain the gains of the previous millennium development goals (MDGs) best child health practices such as improved exclusive breastfeeding, with interventions to reduce the prevalence of prelacteal feeding are not only needed but must also be sustained. EBF is associated with greater reductions in infants risks for specific negative health outcomes, including gastrointestinal and respiratory infections [6]. Breastfeeding practices such as early iniatiation and exclusive breastfeeding is the key practice that can reduce child death and morbidity [7, 8]. Early initiation of breastfeeding within an hour of delivery enhances mother-infant bonding and creates an opportunity for the newborn to receive colostrum with nutritional and protective benefits [9]. It also promotes effective sucking, with successful establishment and maintenance of breastfeeding throughout infancy [10]. In most countries of sub-Sahara Africa, including Nigeria, prelacteal feeding is still prevalent with a significant proportion of mothers offering their newborns various types of prelacteal feeds. The prelacteal feeding rate in Nigeria remains one of the highest in sub-Sahara Africa. It may be a reason why the country has one of the lowest exclusive breastfeeding rates in sub-Sahara Africa. The World Health Organization (WHO) and United Nations Children's Fund (UNICEF) recommends the avoidance of prelacteal feeding within the first six months of life except it is medically indicated [11, 12], because it reduces the chances of a child receiving exclusive breastfeeding. This interaction suggests that the practice of exclusive breastfeeding will be low wherever the prevalence of prelacteal feeding is high as in the case of Nigeria and vice versa. Various prelacteal feeding rate has been reported from different regions of the country. According to the Nigeria Demographic Health survey 2008, geographical variation in the practice of pre-lacteal feeding ranges from 31% in the Southwest to 79% in the Northeast of the country [13]. Studies have shown that the determinants of prelacteal feeding are multi-factorial in nature and vary across regions and cultures. These factors includes antenatal clinic attendance, mode of delivery, place of delivery, type of birth, wealth index, maternal knowledge and beliefs, birth weight, community awareness, community culture, and regions etc [12, 14, 15]. According to the NDHS 2013, a significant number of infants were born in home facilities across communities [16] increasing the risk for prelacteal feeding. However, most previous studies on prelacteal feeding in Nigeria were health facility based, and in urban areas [15, 17]. There is a need to consider the possible influences that may arise from the community, especially for home and other non-facility deliveries which findings from these previous facility-based studies may not explain. Identifying and understanding the effect of the multiple factors contributing to the continuous practice of prelacteal feeding at the community level in our setting is vital to designing interventions to address these problems. This study intended to fill this gap. The objective of this study was to assess the determinants of prelacteal feeding practices among mothers with children aged less than 24 months in the Ile-Ife, Southwest, Nigeria.

## Methods

### Study sites and period

The study was carried out in Ife Central Local Government Area (LGA), Ile-Ife, Osun state, Southwest Nigeria. The state has a population of about 3.4 million people with children under the age of five years estimated to be 684,707 according to the 2006 population census while the study LGA has an estimated population slightly above 167,000 people [18]. There are 11 wards in the selected LGA. The LGA though classified as urban has a mix of rural settlements.

### Study design

A community-based cross-sectional study design supplemented with qualitative data collection using focus group discussion and in-depth interview. Sample size and sampling technique. The sample size was determined using a formula for estimation of single population proportion as follows; using the prevalence of prelacteal feeding in Northern Nigeria as 79% [13], 95% level of confidence, 5% margin of error. A minimum sample size of 255 was obtained. A pre-survey was done for this study to determine which households have the targeted mother-child pairs (0-23 months) before the actual study day. From the surveyed household, two hundred and fifty-five (255) mother with children aged 0-23 months were recruited into this study using a multistage sampling technique. Two wards 4 and 5 were randomly selected among the wards in the LGA through simple random sampling. Enumeration Areas (EAs) in the two selected wards were then assessed. Household listing of the selected EAs was done. Eligible households were then selected through systematic random sampling from all the EAs in the selected wards 4 and 5. One hundred and thirty (130) household were selected in ward 4 while 125 households were selected in ward 5. One mother-child pair (0-23 months) was selected from each household. In households with more than one eligible study subject, only one was selected using simple random selection by balloting system.

### Data collection instrument

Quantitative data collection was done using a structured, pretested, interviewer-administered questionnaire. The questionnaire was designed to meet the objective of the study and adapted partly from the one previously used by UNICEF for CIMCI programme in Nigeria [19]. Applicability, acceptability, and validity of the instrument were evaluated during the pretest. Nine trained data collectors were recruited to assist in data collection. Five were for quantitative data collection while four participated in qualitative data collection. Two days of intensive training on the objective of the study, confidentiality of information, and techniques was conducted. The data collectors were given an interview guide during the training to guide data collection activity. Three focus group discussion (FGD) was done. Two among mothers disaggregated by age into young and old mother, one among grandmother aged over 60 years. Two Key in-depth interviews with two representatives of the ward health committee were also conducted.

### Study variables and operational definitions

The outcome variable was prelacteal feeding defined in this study as giving anything to drink other than human milk to the index child before the initiation of breastfeeding [5]. This operationalised definition differs from the NDHS definition of giving anything to drink other than breast milk in the first three days after delivery [11, 16]. Mothers were asked if they gave any drink other than breast milk to the index child before the initiation of breastfeeding. If the response is *yes* it was coded *1*, and if no it was coded as *0*.

**Figure 1:**
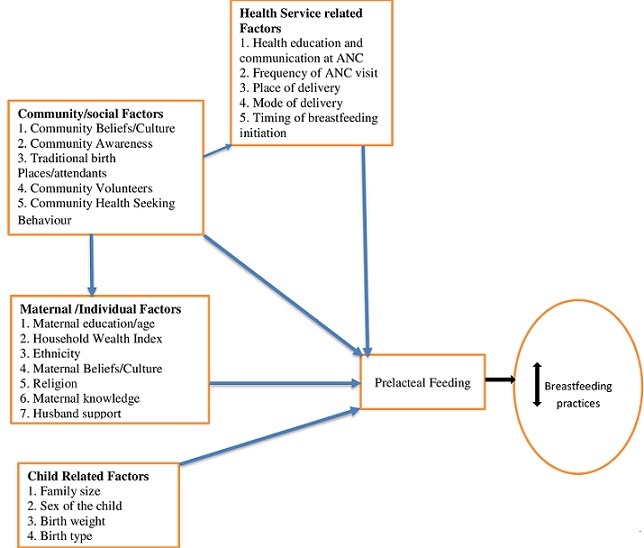
Conceptual framework of determinants of prelacteal feeding and interaction with breastfeeding practices

The independent variable selected for this study were based on reports from previous literature and the conceptual framework which also was designed based on findings from previous researches. These variables were grouped into four factors: community/social level factors (*Belief and cultures, community awareness, community volunteers, community health-seeking behaviours, preponderance of local birth places*), Maternal/individual factors (age, maternal knowledge, husband support, household wealth index educational status, religion, ethnicity), child-related factors (*child's sex, birth order, family size, birth weight*) and health services related factors (place of delivery, mode of delivery, timing of breastfeeding initiation, ante-natal care (ANC) attendance and frequency) (Figure 1). A wealth index was constructed using a composite indicator such as the presence or absence of durable assets in the households etc. Principal component analysis was used to categorise the wealth index as part of an initial comparison study. The wealth index was categorised into lowest, middle and highest. Maternal age was recoded into < 30 and > 31 years, education into marital status (single or married) occupation was recoded into employed and unemployed, family size into ≤4 and >4. Place of delivery was categorised as home delivery and health facility. Mode of delivery into spontaneous vaginal delivery or caesarean section. The number of ANC visits was recoded into 0, 1-3, 4 and above visit.

### Data analysis

#### Quantitative data analysis

Statistical Package for Social Sciences (SPSS Inc., Chicago, IL, USA) version 20 was used for data analysis. Descriptive statistics were used to describe the general characteristics of the study participants. Binary logistic regression analysis was performed to assess the association between each covariate and the outcome variable (prelacteal feeding), the crude odds ratio (COR) with 95% confidence interval was estimated to select variables for the multivariate logistic regression analysis. Variables with p-values of ‹ 0.3 in the binary logistic analysis were entered into the multivariable analysis. Before conducting the multivariate analysis, the independent variables were checked for multicollinearity because of several factors used in the model. Factors considered for the multivariate model were based on the conceptual framework and from findings of previous literature [4,12,14-15]. Adjusted odds ratios (AOR) with their 95% confidence interval (CI) were estimated to assess the strength of association while a p-value ‹0.05 was taken as statistically significant.

#### Qualitative data analysis

Audio recordings of FGD and key in-depth interviews were transcribed by qualitative data experts within 48 hours of the interview to ensure data credibility and dependability. The textual data were then reviewed together and triangulated to reach a better understanding. A content analysis was used to interpret the findings whereby the transcript was read repeatedly and then categorised into words and phrases to find meaningful relationships between the themes that emerged. The results were presented in conjunction with quantitative data.

### Ethical consideration

Ethical clearance was obtained from the Ethics and Research Committee of Obafemi Awolowo University Teaching Hospitals Complex. Written informed consent was given by mothers who consented to participate in the study. Permission was also gotten from the local government authorities.

## Results

### Socio-demographic characteristics

Two hundred and fifty mother-child pairs were involved in the study. The mean (±SD) age of mothers was 29.8 (±5.9) years while those of index children was 14.2 (±7.3) months. Majority of mothers (75.7%) had secondary education and above, most were married, (93.7%) and only 4.3% were housewives. More than half (54.5%) of respondents were in the middle quintiles of the household wealth index (Table 1).

**Table 1:** Socio-demographic characteristics of mothers of children aged less than 24 months in Ile–Ife southwest Nigeria (N= 255)

Variables	Frequency	Percent (%)
**Mothers age (in years)**		
≤30	156	61.2
>31	99	38.8
**Maternal education status**		
No formal and primary	62	24.3
Secondary and above	193	75.7
**Marital status**		
Married	239	93.7
Currently Unmarried	16	6.3
**Maternal occupation**		
Housewife	11	4.3
Not housewife	244	95.7
**Childs Age (months)**		
≤ 6	21	8.2
7-11	102	40.0
12-23	132	51.8
**Childs Sex**		
Male	140	54.9
Female	115	45.1
**Household Wealth quintiles**		
Lowest	73	28.6
Middle	139	54.5
Highest	43	16.9
**Family size**		
≤ 5	96	37.6
≥ 6	159	62.4

### Health service, maternal and community-related characteristics

Ninety-one percent of the mothers attended antenatal care (ANC) clinic in the last pregnancy. Eighty-eight percent received counselling on breastfeeding during ANC clinic in the previous pregnancy. Delivery was attended by a health professional in 86.3% of respondents. Most of the deliveries were through spontaneous vagina delivery (94.9%). Sixty-seven percent attended postnatal/immunization clinic where they again received counseling on breastfeeding (Table 2).

**Table 2:** Health service and community related characteristics of mothers of children aged less than 24 months in Ile-Ife Southwest Nigeria (N = 255)

Variables	Frequency	Percent (%)
**Attended ANC**		
Yes	233	91.4
No	22	8.6
**Number of ANC attended (n=233)**		
≤ 3	24	10.3
≥ 4	209	89.7
**Counseling on breastfeeding at ANC (n= 233)**		
Yes	205	88.0
No	28	12.0
**Delivery attendant**		
Health Professional	220	86.3
Traditional Birth Attendant	35	13.7
**Mode of delivery**		
Vagina delivery	242	94.9
Caesarean section	13	5.1
**Postnatal/ Immunization clinic attendance**		
Yes	171	67.1
No	84	32.9
**Place of delivery**		
Health facilities	220	86.3
Home	35	13.7

### Feeding practices

More than a quarter of mothers (26.3%) reported giving prelacteal feeds to their children. The most common reason for giving prelacteal feeds is the assumption that babies need it for their health (44.8%). A lesser proportion (26.9%) gave prelacteal feed because of delayed lactation, interestingly, (10.4%) did as a result of tradition. Glucose water was the most common type of prelacteal feed given (46.3%), sugar water (25.4%), infant formula (17.9%) and plain water (10.4%). Regarding breastfeeding initiation, (77.6%) of respondents initiated breastfeeding within an hour of delivery. All other mothers (22.4%) did after an hour of delivery or more (Table 3).

**Table 3:** Prelacteal feeding practices among mothers of children aged less than 24 months in Ile-Ife southwest Nigeria (N = 255)

Variables	Frequency	Percent (%)
**Index child given prelacteal feed**		
Yes	67	26.3
No	188	73.7
**Types of prelacteal feeds n=67**		
Sugar water	17	25.4
Glucose water	31	46.3
Infant formula	12	17.9
Plain water	7	10.4
**Reasons for giving prelacteal feeds n=67**		
Delayed lactation	18	26.9
Babies Health need	30	44.8
Tradition	7	10.4
Others*	12	17.9
**Breastfeeding Initiation**		
≤ 1hr	198	77.6
≥ 1 hr	57	22.4

### Factors associated with prelacteal feeding

Binary logistic regression analysis revealed that living in the lowest wealth quintiles, delivery assisted by traditional birth attendants, younger mothers age ≤30 years, giving birth through caesarian section, initiating breastfeeding after one hour of delivery and not attending antenatal clinic, and having below secondary education were statistically associated with prelacteal feeding. However, multivariate logistic regression analysis showed that delivery assisted by traditional birth attendants (p = 0.012), initiating breastfeeding after one hour of delivery (p = 0.002), living in the lowest wealth quintiles (p = 0.003), giving birth through cesarean section (p = 0.002), and not attending antennal clinic (p = 0.001) remained as statistically significant positive predictors of prelacteal feeding practice. The odds of prelacteal feeding was 2.7 times (Adjusted Odds Ratio (AOR): 2.74, 95 % CI 1.43, 5.23) greater among mothers who initiated breastfeeding after one hour compared to those who did within an hour. Mothers who had birth attended by health professional had 25% (AOR=0.75. 95% CI 0.42, 0.97) lower odd of introducing prelacteal feed than mothers who had birth attended by traditional birth attendant. Being in the highest wealth quintiles decreases the odds of prelacteal feeding by 21% (AOR = 0.79, 95% CI 0.51,0.94) compared to middle or lowest wealth quintiles. Likewise, mothers who did not attend antenatal clinic were two and half times (AOR = 2.52, 95% CI 1.05, 5.33) more likely to practice prelacteal feeding than those who attended antenatal clinic (Table 4). The odds of initiating prelacteal feeding was 52% (AOR = 1.52, 95% CI 1.10, 6.34) higher among children delivered via cesarean section compared to those delivered through spontaneous vagina delivery

**Table 4:** Factors associated with prelacteal feeding practice among mothers with children aged less than 24 months in Ile-Ife Southwest Nigeria

Variable	Crude OR(95% CI)	Adjusted OR(95% CI)	P-value
**Maternal occupation**			
Housewife	1.25 (0.89, 2.73)	0.70 (0.47,1.05)	0.382
Not housewife	1		
**Marital status**			
Married	1	1	
Unmarried	1.75 (0.51,5.03)	2.30 (0.76,7.27)	0.274
**Maternal education status**			
Below secondary education	2.15 (1.25,4.15)*	0.76 (0.59,2.92)	0.488
Secondary and above	1	1	
**Maternal age (years)**			
> 31	1	1	
≤ 30	1.61 (1.38,3.99)*	1.29 (0.69,2.49)	0.058
**Mode of Delivery**			
Vagina delivery	1	1	
Caesarean section	1.09 (1.03,4.23)	1.52 (1.10,6.34)*	0.002
Delivery attendant			
Health professional	0.60 (0.52,0.87)	0.75 (0.42,0.97)*	0.012
Traditional birth attendant	1	1	
**Breastfeeding initiation time**			
≤1 hour	1	1	
> 1 hour	2.62 (1.40,4.90)	2.74 (1.43,5.23)*	0.002
**ANC attendance**			
Yes	1	1	
No	1.20 (1.19,1.73)	2.52 (1.05,5.33)*	0.001
**Household wealth quintiles**			
Lowest and middle	1	1	
Highest	0.63 (0.54,0.88)	0.79 (0.51,0.94)*	0.003
**Child sex**			
Male	1	1	
Female	1.35 (0.72, 4.46)	1.20 (0.60,5.52)	0.313

Signifiant P < 0.05

### Findings of the qualitative study

Key themes that emerged from the qualitative study revealed that mothers understood and could define what prelacteal feeding means and were highly awareness of exclusive breastfeeding and its benefits. In spite of this, not all mothers seem to agree fully with the supposed benefits of optimal breastfeeding practices because of beliefs transferred to them by their parents. A 39-year old mother said prelacteal feeding was passed down to us by our parents and the elders cannot be wrong. *Orthodox medicine is just eroding how we were nursed* (FGD). A 60-year old grandmother said we provide prelacteal feed to newborn to meet their health need. For example to improve their digestion and prevent early constipation. Another grandmother opined that *prelacteal foods can prevent infants from disease* (FGD). A 25-year old uneducated young mother pointed out that prelacteal feeding is practiced because some children after exclusive breastfeeding refuse to take other meals and as such initiating them on prelacteal feed will help them appreciate early enough that there are foods other than breast milk (FGD). A 51-year old ward health committee representative however differed in opinion from mothers maintaining that perception on prelacteal feeding varies among households. He was of the opinion that although prelacteal feeding is common, the practice is dependent on the influence of members of households. He also emphasised that household with literate grandparents, in-laws, and families with noticeable support for child health may not practice prelacteal feeding because often times their perception of child care differ due to the influence of formal and health education and family support (In-depth interview).

## Discussion

Pre-lacteal feeding is widely practiced in Nigeria in spite of its association with poor breastfeeding and health outcome. In this study, 26.3% of newborns received prelacteal feed. This is in keeping with the rate (31%) reported in the 2013 NDHS [16] for the Southwest region of the country where the study was conducted and 26.8% found in a study in Ethiopia [20]. However, a higher prevalence (59%) was reported for the entire country [16]. The lower rate in the study area and the Southwest region of the country may be as a result of higher maternal education, maternal health care utilization and increased delivery in health facilities. The common types of prelacteal feeds in the study community were glucose water, sugar water, infant formula (milk) and plain water. This is similar to findings from previous studies [21, 22]. The implication of this is that the types of feed given as prelacteal feeds has not changed just as the practice itself has not reduced significantly. This finding is corroborated by findings from focus group discussion which established that mothers are not unaware of benefits of optimal breastfeeding practices; however, they have a strong belief in practices passed down by their parents and elders in their immediate community. This beliefs influence their perception and contribute to the continuous practice of prelacteal feeding. Behaviour change programmes targeted at reducing the prevalence of prelacteal feeding practices should incorporate not just the dangers of prelacteal feeding but also the risk posed by the types of feeds given to newborns. It should also integrate people in the immediate community of these mothers who influence decisions regarding the care of the newborns such as grandparents, friends of mothers and others. This study found that the odd of prelacteal feeding was lower among mothers who had birth attended by health professional than mothers who had birth attended by traditional birth attendants. This is in keeping with studies from Ethiopia [4] and India [23]. A possible explanation for this is that health professionals are more likely to educate and encourage these mothers on the benefits of optimal breastfeeding and the dangers of prelacteal feeding. In the current study undergoing caesarian section during delivery was significantly associated with a higher likelihood of prelacteal feeding. The higher odd of prelacteal feeding observed among women who had caesarean section compared to spontaneous vaginal deliveries could be linked to the fact that caesarean section often times leads to prolonged maternal-infant separation, pain and discomfort, antibiotics safety concern and occasionally a longer stay in the hospital than anticipated. This finding is in line with reports from previous studies [4, 15, 24]. Antenatal care visit is the best opportunity to promote skilled attendance at birth and to counsel and educate mothers on essential healthy behaviours like newborn feeding [25]. Mothers who attend the antenatal clinic are more likely to be aware of ideal breastfeeding practices that discourage prelacteal feeding. This was true in this study as mothers who did not attend antenatal clinic were two and half times more likely to practice prelacteal feeding. A high percentage of women in this study attended the antenatal clinic, which suggests that antennal clinic use in the community of study might likely be high. The effect is that the higher the community antenatal clinic use the more likely the chance of developing a norm that discourages prelacteal feeding. Late initiation of breastfeeding was also associated with increased odd of prelacteal feeding. The odds of giving prelacteal feed in this study was approximately three times higher among mothers who initiated breastfeeding after one hour of delivery compared to mothers who did within an hour of delivery. The finding is in congruence with studies done previously [4, 14, 26]. It is possible that as the interval between delivery and breastfeeding initiation increases, there will be more opportunity for family and cultural influences that promote wrong feeding practices such as prelacteal feeding. In this study, living in the highest wealth quintiles was positively associated with the introduction of prelacteal feeding. The odd of prelacteal feeding was lower among those in the higher wealth quintiles compared to those in the middle or lowest wealth quintiles. The positive effect of wealth in improving mother's health-seeking behaviour and the utilization of maternal health care services may account for this finding. This finding is supported by a report from the key in-depth interview which suggested that households with literate families and noticeable support for child health often do not engage in prelacteal feeding. Mothers in the lowest wealth quintiles were less likely to have antenatal care and attend institutional delivery and as such may not have access to appropriate health information and counselling that can improve their child health practices. This finding is similar to those of Tariku *et al.* in Ethiopia [20]; however, it contradicts findings from some other studies [4, 12]. The strength of this study draws from the qualitative component which established a strong link between community beliefs, maternal influences and the practice of prelacteal feeding. It also revealed pertinent determinants of prelacteal feeding. However, the study is not without limitations; the study limitation relates to the fact that the information obtained from the mothers is subject to recall bias. Besides, due to the cross-sectional study design, caution must be exercised in making causal inferences of the identified determinants of prelacteal feeding.

## Conclusion

Prelacteal feeding was prevalent in the study community. This high prevalence was significantly associated with maternal, health service and community-related factors such as low household wealth status, delivery through caesarian section, having birth assisted by traditional birth attendants, late initiation of breastfeeding and poor antenatal clinic attendance. Community beliefs and practices was a major underlying factor for prelacteal feeding practice. Intervention that strengthens individual and community access to appropriate health information and maternal health services are necessary for reducing prelacteal feeding practices.

### What is known about this topic

- Prelacteal feeding has been reported to be highly prevalent and widely practiced in the country;
- Regional variations in the prevalence of prelacteal feeding exist in the country.

### What this study adds

- The study reveals that individual factors such as low household wealth status, community and health service related factors like caesarian section, poor ANC attendance, patronage of traditional birth attendants, etc. contribute to the practice of prelacteal feeding;
- The study establishes community beliefs and practices as an underlying factor for prelacteal feeding practice.

## Competing interests

The authors declare no competing interests.

## References

[1] Meshram I, Laxmaiah A, Venkaiah k, Brahamam GN (2012). Impact of feeding and breastfeeding practices on the nutritional status of infants in a district of Andhra Pradesh. India Natl Med J. India.

[2] (2011). Central Statistical Agency (CSA) Ethiopia: Demographic and Health Survey 2011.

[3] Lakati A, Makokha O, Binns C, Kombe Y (2010). The effect of pre-lacteal feeding on full breastfeeding in Nairobi, Kenya. East Afr J Public Health.

[4] Belachew AB, Kahsay AB, Abebe YG (2016). Individual and community-level factors associated with introduction of prelacteal feeding in Ethiopia. Arch Public Health.

[5] WHO/UNICEF (2009). Baby-friendly hospital initiative (BFHI). Revised, updated and expanded for integrated care. Section 3, Breastfeeding promotion and support in a baby-friendly hospital: a 20-hour course for maternity staff.

[6] Kramer MS, Kakuma R (2012). Optimal duration of exclusive breastfeeding. Cochrane Database Syst Rev.

[7] Victora CG, Bahl R, Barros AJD, Franca GVA, Horton S, Krasevec J (2016). Breastfeeding in the 21^st^century: epidemiology, mechanisms, and lifelong effect. Lancet.

[8] Godhia ML, Patel N (2013). Colostrum - its Composition, benefits as a Nutraceutical - A Review. Curr Res Nutr Food Sci.

[9] Himani, Kaur B, Kumar P (2011). Effect of initiation of breastfeeding within one hour of the delivery on maternal-infant bonding?. Nurs Midwifery Res J.

[10] Begum K, Dewey KG (2010). Impact of early initiation of exclusive breastfeeding on newborn deaths. Alive and Thrive Technical Brief.

[11] WHO and UNICEF (1990). Innocenti declaration on the protection, promotion and support of breastfeeding. Proceedings of the Breastfeeding in the 1990s: a global initiative at spedale Degli Innocenti, Florence, Italy, 30th July-1st August 1990.

[12] Khanal V, Adhikari M, Sauer K, Zhao Y (2013). Factors associated with the introduction of prelacteal feeds in Nepal: findings from the Nepal demographic and health survey 2011. Int Breastfeed J.

[13] UNICEF Situation Analysis of Children and Women in Nigeria.

[14] Legesse M, Demena M, Mesfin F, Haile D (2014). Prelacteal feeding practices and associated factors among mothers of children aged less than 24 months in Raya Kobo district, North Eastern Ethiopia: a cross-sectional study. Int Breastfeed J.

[15] Ibadin OM, Ofili NA, Monday P, Nwajei CJ (2013). Prelacteal feeding practices among lactating mothers in Benin City, Nigeria. Niger J Paed.

[16] National Population Commission, Federal Republic of Nigeria (2013). Final Report on Nigeria Demographic and Health Survey 2013.

[17] Adetunji O, Joseph A, Olusola E, Joel-Medewase V, Fadero F, Oyedeji G (2006). Pre-lacteal feeding practices of doctors and nurses in a state and teaching hospital in western Nigeria: A cause for concern. Int J Nutr Wellness.

[18] National Population Commission (2009). Nigeria Demographic and Health Survey 2008.

[19] United Nations Children's Fund/Federal Ministry of health (2005). IMCI in the Hands of Families: Nigeria Country Report of Baseline Studies on Key Family and Community Practices in IMCI-implemented LGAs.

[20] Tariku A, Biks GA, Wassie MM, Gebeyehu A, Getie AA (2016). Factors associated with prelacteal feeding in the rural population of northwest Ethiopia: a community cross-sectional study. Int Breastfeed J.

[21] Berde AS, Yalcin SS, Ozcebe H, Uner S, Caman OK (2017). Determinants of pre-lacteal feeding practices in urban and rural Nigeria; a population-based cross-sectional study using the 2013 Nigeria demographic and health survey data. Afr Health Sci.

[22] Lakati AS, Makokha OA, Binns CW, Kombe Y (2010). The effect of pre-lacteal feeding on full breastfeeding in Nairobi, Kenya. East Afr J Public Health.

[23] Shah BD, Dwivedi LK (2013). Newborn care practices: a case study of tribal women, Gujarat. Health.

[24] El-Gilany AH, Abdel-Hady DM (2014). Newborn first feed and prelacteal feeds in Mansoura, Egypt. Biomed Res Int.

[25] U.S Agency for International Development (USAID) (2007). Focused ANC: providing\integrated, individualized care during pregnancy.

[26] Egata G, Berhane Y, Worku A (2013). Predictors of non-exclusive breastfeeding at 6 months among rural mothers in east Ethiopia: a community-based analytical cross-sectional study. Int Breastfeed J.

